# Lionfish (*Pterois* spp.) invade the upper-bathyal zone in the western Atlantic

**DOI:** 10.7717/peerj.3683

**Published:** 2017-08-17

**Authors:** Erika Gress, Dominic A. Andradi-Brown, Lucy Woodall, Pamela J. Schofield, Karl Stanley, Alex D. Rogers

**Affiliations:** 1 Nekton Foundation, Begbroke Science Park, Begbroke, Oxfordshire, United Kingdom; 2 Department of Zoology, University of Oxford, Oxford, Oxfordshire, United Kingdom; 3 Operation Wallacea, Old Bolingbroke, Lincolnshire, United Kingdom; 4 U.S. Geological Survey, Gainesville, FL, United States of America; 5 Roatan Institute of Deepsea Exploration, West End, Roatan, Honduras

**Keywords:** Lionfish, *Pterois*, Deep sea, Bermuda, Roatan Honduras, Invasive species, Upper bathyal, Depth record

## Abstract

Non-native lionfish have been recorded throughout the western Atlantic on both shallow and mesophotic reefs, where they have been linked to declines in reef health. In this study we report the first lionfish observations from the deep sea (>200 m) in Bermuda and Roatan, Honduras, with lionfish observed to a maximum depth of 304 m off the Bermuda platform, and 250 m off West End, Roatan. Placed in the context of other deeper lionfish observations and records, our results imply that lionfish may be present in the 200–300 m depth range of the upper-bathyal zone across many locations in the western Atlantic, but currently are under-sampled compared to shallow habitats. We highlight the need for considering deep-sea lionfish populations in future invasive lionfish management.

## Introduction

Non-native lionfish, first documented in the western Atlantic region in the 1980s (Schofield, 2009; Schofield, 2010), are considered a major threat to western Atlantic reef communities (Sutherland et al., 2010). Lionfish are benthic generalist predators, and their presence on shallow coral reefs has been associated with up to 65% decline in their prey fish biomass (Green et al., 2012), leading to overall declines in fish recruitment of up to 79% (Albins & Hixon, 2008). In some cases lionfish have been observed to feed on critically-endangered reef fish (Rocha et al., 2015). On both shallow reefs and mesophotic coral ecosystems (MCEs, reefs from 30 to approximately 150–180 m depth; (Hinderstein et al., 2010)), non-native lionfish are thought to cause increased algal cover by consuming herbivores and causing trophic cascades (Lesser & Slattery, 2011; Slattery & Lesser, 2014; Kindinger & Albins, 2017). Native to the Indian and Pacific oceans and Red Sea, lionfish in the western Atlantic have now been recorded from New York, USA in the north (Meister et al., 2005), to as far south as the southeastern coast of Brazil (Ferreira et al., 2015). In addition, there is a second lionfish invasion currently underway in the Mediterranean Sea (Kletou, Hall-Spencer & Kleitou, 2016). Two species of non-native lionfish have been recorded in the western Atlantic: *Pterois volitans* (Linnaeus, 1758) and *P. miles* (Bennett, 1828) (Hamner, Freshwater & Whitfield, 2007), though they are believed to be ecologically synonymous in their impacts to western Atlantic marine communities (Morris et al., 2009).

The majority of research on lionfish invasions has focused on shallow coral reefs (<30 m), mangroves and seagrass beds (Morris et al., 2009; Claydon, Calosso & Traiger, 2012). However, recent studies have highlighted their widespread presence on MCEs across the western Atlantic invaded range (Andradi-Brown et al., 2017a), which is unsurprising, as they have been recorded on MCEs in many locations in their native range. For example, *P. miles* at 65 m in the Red Sea (Brokovich et al., 2008), and *P. volitans* at 75 m in New Caledonia (Kulbicki et al., 2012), 61 m in Micronesia (Andradi-Brown et al., 2017a), 61 m in the Philippines (Andradi-Brown et al., 2017a), and at 80 m in American Samoa (Wright, 2005). With two exceptions (see next paragraph), MCEs represent the deepest depths lionfish have been previously reported from in the western Atlantic. For example, from remote operated vehicle (ROV) surveys: 112 m in the northwestern Gulf of Mexico (Nuttall et al., 2014), 100 m off North Carolina, USA (Meister et al., 2005), 126 m on the Desecheo Ridge west of Puerto Rico (Quattrini et al., 2017), and 167 m on the Conrad Seamount in the Anegada Passage (Quattrini et al., 2017). Lionfish have also been observed at 120 m from submersible dives in Honduras (Schofield, 2010), and collected from trawl surveys >80 m depth in the eastern Gulf of Mexico (Switzer et al., 2015). In addition, diver-based surveys on MCEs have reported sightings in the 30–100 m range in Puerto Rico (Bejarano, Appeldoorn & Nemeth, 2014), Bermuda (Pinheiro et al., 2016), and the Lesser Antilles (De León et al., 2013). Therefore, it has been suggested that lionfish have widely colonised MCEs across the western Atlantic (Andradi-Brown et al., 2017a).

In August 2010, while conducting submersible surveys off Lyford Cay, Nassau, The Bahamas, lionfish were observed at 300 m (pers. comm. from RG Gilmore in: Albins & Hixon, 2013; McGuire & Hill, 2014). While in Curaçao, the *Curasub* has reported observing lionfish regularly down to 247 m depth (Tornabene & Baldwin, 2017). To our knowledge these sightings represent the maximum known depth distribution of lionfish in the western Atlantic, and the only records of lionfish in the deep sea (defined as >200 m depth; Rogers, 2015). It is not clear whether these sightings represent isolated incidents of lionfish reaching these depths, or whether lionfish more regularly use habitats in the >200 m depth range, but they have not previously been recorded because of limited surveys within this depth range.

**Figure 1:**
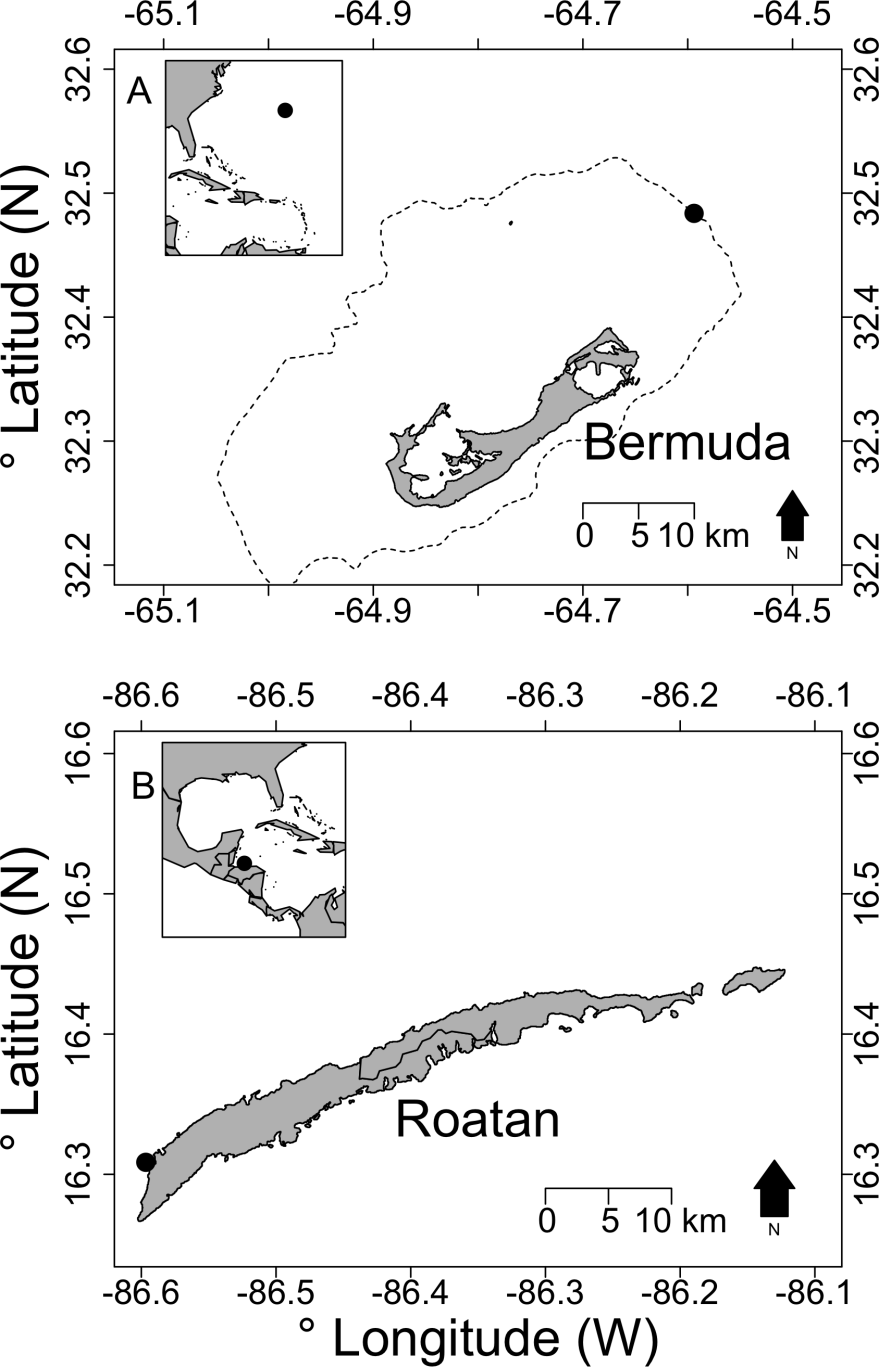
Map showing location of deepest observed lionfish for (A) Bermuda and (B) Roatan, Honduras. Inset maps indicate the locations of Bermuda and Roatan respectively relative to the western Atlantic region. In (A) the dashed line indicates the 50 m depth contour to show the outline of the Bermuda platform. The reef drops off steeply at this location, such that our 304 m lionfish observation is close to the 50 m depth contour.

In this study we report visual observations of lionfish >200 m depth in two new locations within the western Atlantic region: Bermuda and Roatan, Honduras. We also consider other lionfish records that could potentially indicate that lionfish may be more widespread at >200 m depth across the western Atlantic range.

## Methods

Bermuda is a series of islands located far off the continental shelf in the northwestern Sargasso Sea (Fig. 1A). The islands exist on a large shallow-water platform (approximately 20 m depth, 623 km^2^ area) which are the eroded remains of a Meso-Cenozoic volcanic peak (Coates et al., 2013). The platform is surrounded by a shallow slope, which transitions into near-vertical walls at around 100 m (Coates et al., 2013). While deep reef areas of Bermuda are poorly studied, with few observations below mesophotic depths, there are established MCE communities around Bermuda to at least 80 m (Pinheiro et al., 2016). MCE to deep-sea benthic organisms and benthic-associated fish surveys were undertaken during daylight hours using the *Nemo* and *Nomad* Triton 1000-2 class submersibles (Vero Beach, Florida, USA) down to 300 m depth around the edge of the Bermuda platform during July and August 2016 as part of the Nekton Foundation/XL-Catlin Deep-Ocean Survey –Mission 1 (www.nektonmission.org). In total, 17 dives were conducted to 300 m between both submersibles. Research permits for Bermuda were issued by the Department of Environment and Natural Resources, Bermuda (No. 2016070751).

In contrast, Roatan is an island in the Caribbean Sea located off the north coast of mainland Honduras (Fig. 1B). Roatan is approximately 50 km long and 2–4 km wide, and has a total land area of about 200 km^2^. This island is surrounded by shallow fringing coral reefs, which transition into MCEs at increased depths. The Roatan Institute of Deepsea Exploration conducts commercial submarine tourism, using the *Idabel* submarine allowing tourists to observe deep-sea habitats to 610 m depth. With year-round operations from Half Moon Bay, West End, Roatan, *Idabel* conducted 224 dives ≥300 m between Jan 2015–April 2017. During March 2017 visual observations of benthic communities and their associated fish communities were conducted on a night dive to 300 m depth. Visual/video lionfish observations in Roatan were covered under the Roatan Institute of Deepsea Exploration operating permit issued by the Municipalidad de Roatan (No. 1391).

To identify other records of deep lionfish we examined 6,814 lionfish records from the US Geological Survey Nonindigenous Aquatic Species database (USGS-NAS, 2017). Lionfish records in the database have been gathered from media reports, scientific publications and direct reports to the database managers. All records contain a GPS location, and in some cases a short description of the conditions under which the lionfish was observed and/or a photo of the lionfish. In some cases the descriptions accompanying records included depth information, though this is not formatted in a consistent way (for example using different units such as metres, feet, fathoms) and contained within a larger text record description. We initially viewed these descriptions to identify any records directly stating lionfish observations at depths ≥200 m, converting any depth information provided into metres for consideration. To further identify potential lionfish records from ≥200 m depth, we downloaded the 2014 General Bathymetric Chart of the Oceans (http://www.gebco.net) 30 arc-second interval grid bathymetry for the western Atlantic region. We used the raster package (Hijmans, 2015) in R (R Core Team, 2013) to identify approximate depths of all lionfish records based on GPS location. All records associated with bathymetry ≥200 m depth were individually reviewed and classified as potential deep-sea individuals, or excluded. Records were excluded for any of the following reasons: (i) specific depth information was available in the record indicating the fish was <200 m depth, (ii) the record reports that the observation was made by a diver or snorkeler, (iii) the location of the record is a well known/established shallow reef diving/snorkelling site, or (iv) the lionfish were collected by hook-and-line making it highly unlikely they were from ≥200 m depth. Raw data are available from the US Geological Survey Nonindigenous Aquatic Species database (USGS-NAS, 2017), and ESM1 contains a list of all lionfish GPS locations present in the database at the time of analysis that were used in this study. ESM2 contains the raw R code used to assign a depth to each lionfish record.

## Results

**Figure 2:**
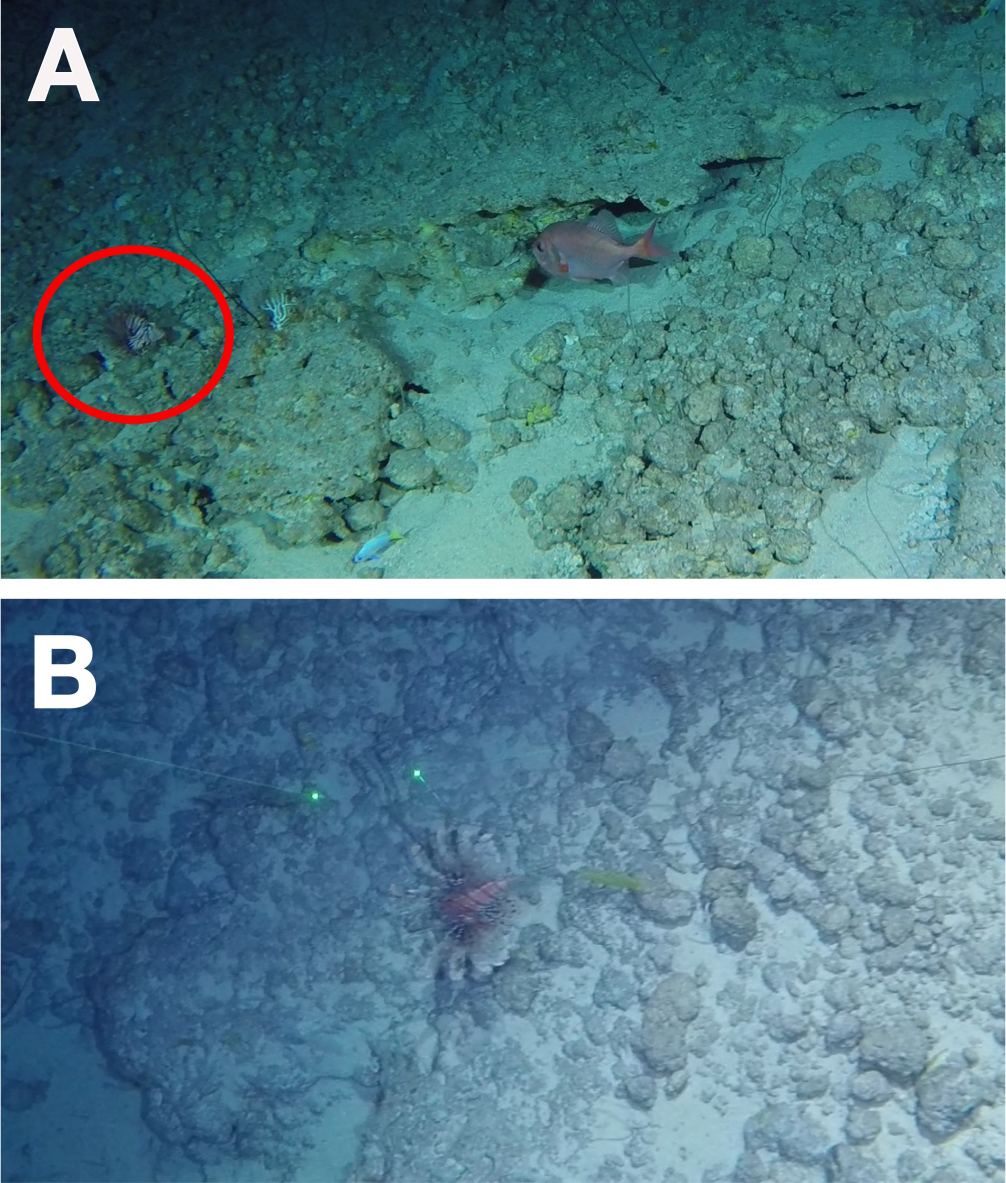
Lionfish at 297 m depth off the northeastern slope of the Bermuda platform. (A) The lionfish resting on the reef is indicated within the red circle. Other fish species shown are *Gephyroberyx darwinii* and cf. *Pronotogrammus martinicensis*. (B) Lionfish swimming over the benthos. The laser dots are separated by 0.25 m. Both (A) and (B) show the same individual that swam across the benthos as disturbed by the submersible.

In Bermuda during daytime dives on 28 July 2016 off the northeastern edge of the Bermuda platform at 32.483683 N, 64.59395 W (Fig. 1A; GPS coordinates in WGS84 format), multiple lionfish were observed. The deepest lionfish were a single individual observed at 304 m depth, and another individual at 297 m (Fig. 2). Water temperature was recorded on the submersible during the dive as 19.7 °C at 300 m. The laser points in Fig. 2B are 0.25 m apart, suggesting an approximate total length of 21 cm for this individual at 297 m.

**Figure 3:**
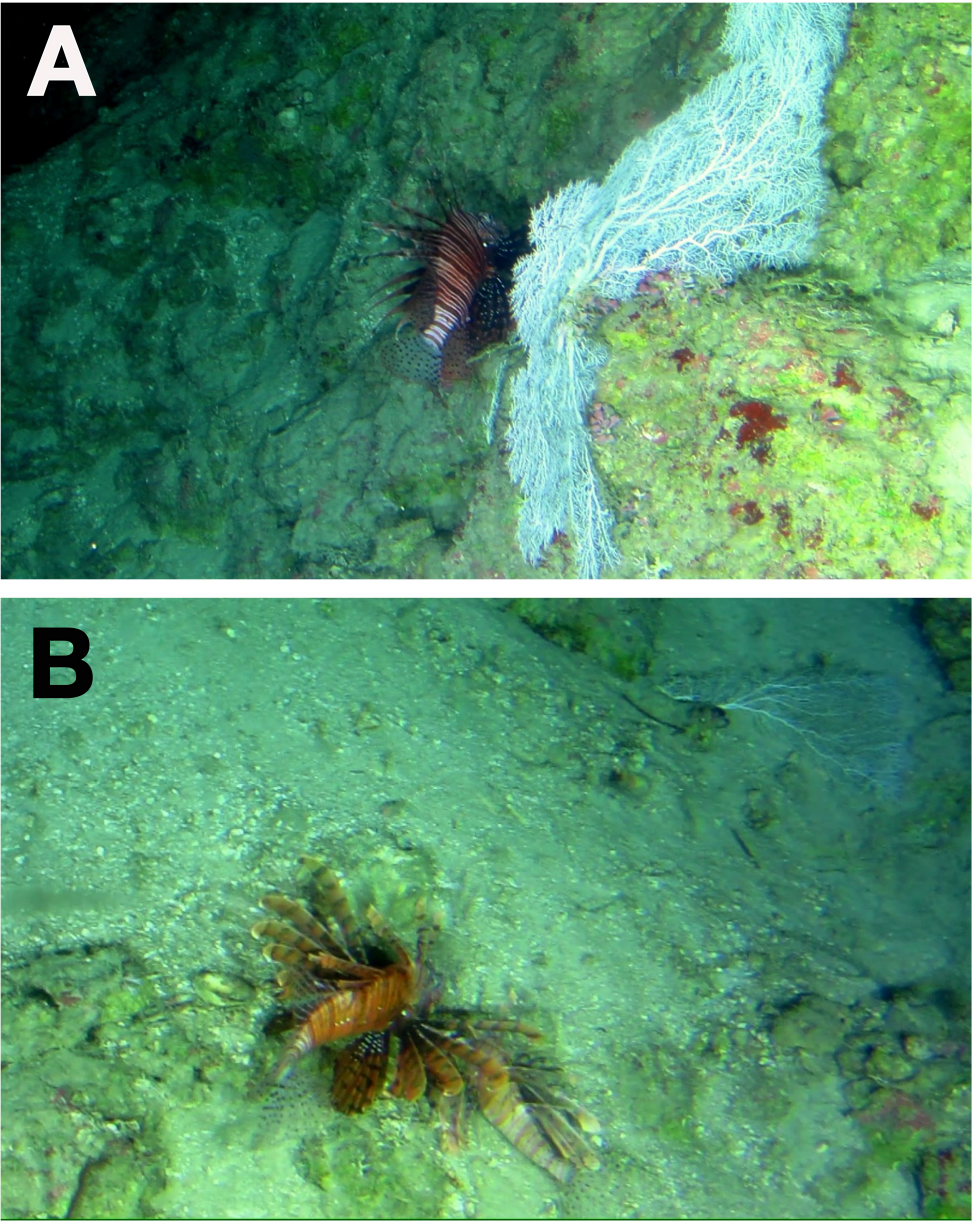
Lionfish off Half Moon Bay, West End, Roatan, Honduras. (A) Lionfish swimming over the benthos at 180 m depth, and (B) two lionfish resting at 240 m depth.

In Roatan, on 11 March 2017 off Half Moon Bay, West End at 16.308565 N, 86.596681 W (Fig. 1B) five lionfish were observed and photographed down to a depth of 240 m (Fig. 3). Individuals were seen in on lower-MCEs (180 m; Fig. 3A), and the upper-bathyal (240 m; Fig. 3B). Water temperature was recorded on this dive as approximately 15 °C at 240 m. However, with year-round tourist submarine dives operating from Half Moon Bay visiting deep reef habitats ≥300 m (224 dives between Jan 2015–April 2017), the *Idabel* has regularly observed lionfish to a maximum depth of 250 m.

**Figure 4:**
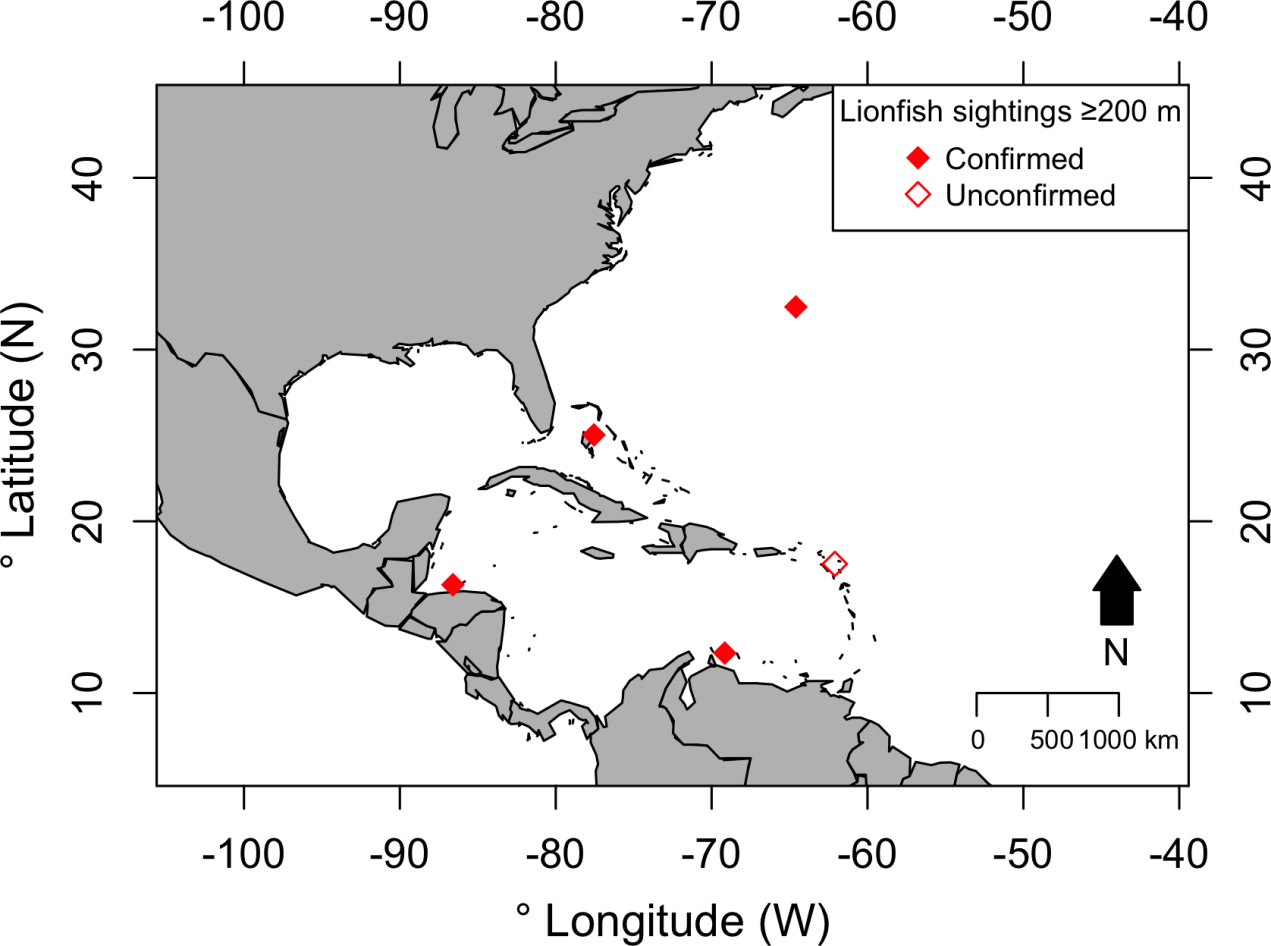
Locations of confirmed and possible lionfish observations ≥200 m depth in the western Atlantic. The confirmed sightings represent our observations in Bermuda and Roatan, and the previously reported observations in the Bahamas (Albins & Hixon, 2013) and Curaçao (Tornabene & Baldwin, 2017). The unconfirmed sighting represent a record from the US Geological Survey Nonindigenous Aquatic Species database associated with bathymetry ≥200 m, though there is no direct information on the depth of the lionfish observation for this record.

When analysing records from the US Geological Survey Nonindigenous Aquatic Species database, no records were found explicitly stating a depth of observation ≥200 m. However, 186 records out of the 6,814 records were associated with bathymetry ≥200 m. Of these, after scrutinising the text descriptions, we excluded 185 records as being too shallow. Many of these records represented sites with steep walls spanning from shallow reefs to >200 m depth, and while the resolution of the available bathymetry suggested these were ≥200m, when checking the associated meta-data for these 185 records it clearly indicated that the lionfish were most-likely <200 m. The one record that we retained did not contain enough detail to confirm or reject it as a sighting from >200 m. Figure 4 shows the locations of this unconfirmed record, the previously confirmed 300 m lionfish observation in the Bahamas (Albins & Hixon, 2013; McGuire & Hill, 2014), the recorded observations in Curaçao at 247 m (Tornabene & Baldwin, 2017), and the locations of our deep-sea lionfish observations in Bermuda and Roatan. We have now added our new deep-sea lionfish observations to the US Geological Survey Nonindigenous Aquatic Species database.

## Discussion

In this study we report deep-sea lionfish observations from the upper-bathyal zone in two new locations within the invaded western Atlantic lionfish range. Both in Bermuda, close to the northern limit of the lionfish overwintering invaded range (Eddy et al., 2016), and in Roatan, within the centre of the lionfish invaded range (Schofield, 2010), we report lionfish >200 m depth. Because of the large geographical distance between our observations, combined with the previous confirmed observations of lionfish >200 m from the Bahamas (pers. comm. from R.G. Gilmore in: Albins & Hixon, 2013) and Curaçao (Tornabene & Baldwin, 2017), we suggest that lionfish may be more widespread and common than presently understood in deep-sea habitats in the 200–300 m depth range and that this deeper aspect of the lionfish invasion has likely been under-sampled.

When searching the US Geological Survey Nonindigenous Aquatic Species database, we found one lionfish record located over bathymetry ≥200 m without stating a depth or giving any indication of depth. While the US Geological Survey Nonindigenous Aquatic Species records have been placed over bathymetry in previous studies, leading to the suggestion that lionfish may extend their maximum depth to 610 m (Johnston & Purkis, 2011), our results indicate depth records generated in this way must be treated with caution. The grid resolution of bathymetry available at a regional level is not sufficient to generate precise lionfish depth information over undersea structures such as walls and steep slopes, where large differences in depth occur within one raster grid square. For this reason, despite identifying 186 records associated with deeper bathymetry, 185 of these were excluded for containing either specific depth details or enough information to suggest that they were most likely shallower reef or MCE observations. Some of these excluded observations were from lionfish associated with oil and gas rigs, where lionfish were associated with the rig structure at shallower depths rather than actually with seabed benthic habitats. Therefore, from simply matching GPS locations with bathymetry, these records would appear to be >200 m and far from any shallower habitat, yet they actually represent shallower lionfish. As many of these records in the US Geological Survey Nonindigenous Aquatic Species database come from recreational and scientific divers and fisheries, we would expect these records to be biased towards shallow reefs where the majority of sampling has occurred. Therefore, it was not surprising that 6,628 of the 6,814 records were associated with shallow reef or MCE bathymetry. With this biased survey effort to the shallows, the lack of records >200 m in the database should be treated as an indication of under-sampling at depth, and not that lionfish are not present in the upper-bathyal.

It is not clear why differences in the maximum depth of observations exist between Roatan, our observations in Bermuda and previous observations in the Bahamas and Curaçao. While in Bermuda we observed lionfish to the maximum survey depth (304 m), in Roatan, despite 224 submarine dives to ≥300 m over the past 2.3 years, lionfish have not been observed deeper than 250 m. There are many possible explanations related to changing environmental conditions, such as temperature and light, or availability of prey. For example, lionfish are limited by temperature (Whitfield et al., 2014; Dabruzzi, Bennett & Fangue, 2017), with lab experiments suggesting they are unable to survive temperatures <10 °C, but crucially they ceased feeding at temperatures <16.1 °C (Kimball et al., 2004). While detailed temperature data across the depth gradient is not available for the locations we surveyed, water temperature was approximately 15 °C at 240 m in Roatan when we photographed lionfish in March 2017. Therefore, it is possible that the 250 m maximum depth of lionfish observations around Roatan may be caused by temperature limitation. In contrast, water temperature was 19.7 °C in Bermuda at 300 m, above the temperature of feeding cessation for lionfish (Kimball et al., 2004). This suggests that if temperature is the main limiting factor for maximum depth, we may expect lionfish to extend even deeper than 304 m in Bermuda.

Other factors such as light could also influence the maximum depth for lionfish. Lionfish are visual predators (Cure et al., 2012); therefore, despite previous studies indicating reef fish have high visual system plasticity to adapt to low light levels at depth (Brokovich et al., 2010), it is likely they will be limited by light. Bermuda has high light penetration (Fricke & Meischner, 1985; Coates et al., 2013), while Roatan suffers from higher sedimentation rates (Mehrtens et al., 2001; Harborne, Afzal & Andrews, 2001), likely reducing light penetration to lower levels than Bermuda. Further research is required to understand the ecological and physiological constraints on maximum lionfish depths.

Little is known about the potential impacts of invasive lionfish on the upper-bathyal zone. However, shallow reef research has suggested large declines in native reef fish abundance and recruitment are caused by lionfish (Albins & Hixon, 2008; Green et al., 2012). Shallow reef fish species generally have higher individual and population growth rates when compared to deep sea fish species (Rogers, 1994; Norse et al., 2012). Therefore, predation by lionfish may have greater potential for damage to native fish communities in the upper-bathyal zone. With so few records of bathyal lionfish and no quantitative estimates of lionfish densities, the ecological impacts at >200 m depth is unknown.

Current lionfish management is highly biased towards shallow reef habitats, with diver-conducted culling the major control measure implemented in the western Atlantic (Morris et al., 2009). While shallow reef culling has been found to reduce lionfish densities (Frazer et al., 2012) and help native fish populations recover (Green et al., 2014), a recent study has suggested strong depth-specific effects of culling on lionfish densities, with substantial lionfish populations remaining on MCEs despite shallow culling (Andradi-Brown et al., 2017b). Previous modelling studies have highlighted that substantial deep refuges for lionfish have the potential to undermine current management programmes (Arias-González et al., 2011). Therefore, if lionfish are widespread in the 200–300 m depth range across the western Atlantic this raises further challenges for lionfish management. There are currently few effective methods for lionfish removal in water too deep for diving, with trapping being the only widely used method. In Bermuda, lobster traps have been used to remove lionfish from MCEs, with trap modifications substantially reducing bycatch of other fish species (Pitt & Trott, 2015). Though measures of trapping effectiveness for reducing deep lionfish populations are still lacking. Traps could be trialled deeper for lionfish control in the 200–300 m range, and cameras used to monitor effects on lionfish densities.

This study documents non-native lionfish in the upper-bathyal zone in Bermuda, and Roatan, Honduras for the first time. Our observations, combined with other lionfish records, suggest that lionfish could potentially be present in 200–300 m depth habitat in many locations in the western Atlantic. Further surveys should be conducted to assess how widely lionfish are using upper-bathyal habitats, and to establish their population densities. Our results highlight the need to consider deeper lionfish populations in management programmes.

##  Supplemental Information

10.7717/peerj.3683/supp-1 Supplemental Information 1 Lionfish GPS locations

10.7717/peerj.3683/supp-2 Supplemental Information 2 R script to assign a depth to each lionfish record

## Additional Information and Declarations

### Data Availability

The following information was supplied regarding data availability: All raw data required for this study is contained in the Supplemental Files.

## References

[ref-1] Albins MA, Hixon MA (2008). Invasive Indo-Pacific lionfish *Pterois volitans* reduce recruitment of Atlantic coral-reef fishes. Marine Ecology Progress Series.

[ref-2] Albins MA, Hixon MA (2013). Worst case scenario: potential long-term effects of invasive predatory lionfish (Pterois volitans) on Atlantic and Caribbean coral-reef communities. Environmental Biology of Fishes.

[ref-3] Andradi-Brown DA, Grey R, Hendrix A, Hitchner D, Hunt C, Gress E, Madej K, Parry R, Régnier-McKellar C, Jones O, Arteaga M, Izaguirre A, Rogers AD, Exton DA (2017b). Depth-dependent effects of culling—do mesophotic lionfish populations undermine current management?. Royal Society Open Science.

[ref-4] Andradi-Brown DA, Vermeij MJA, Slattery M, Lesser M, Bejarano I, Appeldoorn R, Goodbody-Gringley G, Chequer AD, Pitt JM, Eddy C, Smith SR, Brokovich E, Pinheiro HT, Jessup ME, Shepherd B, Rocha LA, Curtis-Quick J, Eyal G, Noyes TJ, Rogers AD, Exton DA (2017a). Large-scale invasion of western Atlantic mesophotic reefs by lionfish potentially undermines culling-based management. Biological Invasions.

[ref-5] Arias-González JE, González-Gándara C, Luis Cabrera J, Christensen V (2011). Predicted impact of the invasive lionfish *Pterois volitans* on the food web of a Caribbean coral reef. Environmental Research.

[ref-6] Bejarano I, Appeldoorn RS, Nemeth M (2014). Fishes associated with mesophotic coral ecosystems in La Parguera, Puerto Rico. Coral Reefs.

[ref-7] Brokovich E, Ben-Ari T, Kark S, Kiflawi M, Dishon G, Iluz D, Shashar N (2010). Functional changes of the visual system of the damselfish *Dascyllus marginatus* along its bathymetric range. Physiology & Behavior.

[ref-8] Brokovich E, Einbinder S, Shashar N, Kiflawi M, Kark S (2008). Descending to the twilight-zone: changes in coral reef fish assemblages along a depth gradient down to 65 m. Marine Ecology Progress Series.

[ref-9] Claydon J, Calosso MC, Traiger SB (2012). Progression of invasive lionfish in seagrass, mangrove and reef habitats. Marine Ecology Progress Series.

[ref-10] Coates KA, Fourqurean JW, Kenworthy WJ, Logan A, Manuel SA, Smith SR, Sheppard CRC (2013). Introduction to Bermuda: geology, oceanography and climate. Coral Reefs of the United Kingdom Overseas Territories, Coral Reefs of the World 4.

[ref-11] Cure K, Benkwitt CE, kinson, Pickering EA, Pusack TJ, McIlwain JL, Hixon MA (2012). Comparative behavior of red lionfish *Pterois volitans* on native Pacific versus invaded Atlantic coral reefs. Marine Ecology Progress Series.

[ref-12] Dabruzzi TF, Bennett WA, Fangue NA (2017). Thermal ecology of red lionfish *Pterois volitans* from southeast Sulawesi, Indonesia, with comparisons to other Scorpaenidae. Aquatic Biology.

[ref-13] De León R, Vane K, Bertuol P, Chamberland VC, Simal F, Imms E, Vermeij M (2013). Effectiveness of lionfish removal efforts in the southern Caribbean. Endangered Species Research.

[ref-14] Eddy C, Pitt J, Morris Jr JA, Smith S, Goodbody-Gringley G, Bernal D (2016). Diet of invasive lionfish (*Pterois volitans* and *P. miles*) in Bermuda. Marine Ecology Progress Series.

[ref-15] Ferreira CEL, Luiz OJ, Floeter SR, Lucena MB, Barbosa MC, Rocha CR, Rocha LA (2015). First record of invasive lionfish (*Pterois volitans*) for the Brazilian Coast. PLOS ONE.

[ref-16] Frazer TK, Jacoby CA, Edwards MA, Barry SC, Manfrino CM (2012). Coping with the lionfish invasion: can targeted removals yield beneficial effects?. Reviews in Fisheries Science.

[ref-17] Fricke H, Meischner D (1985). Depth limits of Bermudan scleractinian corals: a submersible survey. Marine Biology.

[ref-18] Green SJ, Akins JL, Maljković A, Côté IM (2012). Invasive lionfish drive Atlantic coral reef fish declines. PLOS ONE.

[ref-19] Green SJ, Dulvy NK, Brooks AML, Akins JL, Cooper AB, Miller S, Côté IM (2014). Linking removal targets to the ecological effects of invaders: a predictive model and field test. Ecological Applications.

[ref-20] Hamner RM, Freshwater DW, Whitfield PE (2007). Mitochondrial cytochrome b analysis reveals two invasive lionfish species with strong founder effects in the western Atlantic. Journal of Fish Biology.

[ref-21] Harborne AR, Afzal DC, Andrews MJ (2001). Honduras: Caribbean Coast. Marine Pollution Bulletin.

[ref-22] Hijmans RJ (2015). https://cran.r-project.org/web/packages/raster/index.html.

[ref-23] Hinderstein LM, Marr JCA, Martinez FA, Dowgiallo MJ, Puglise KA, Pyle RL, Zawada DG, Appeldoorn R (2010). Theme section on “Mesophotic coral ecosystems: characterization, ecology, and management”. Coral Reefs.

[ref-24] Johnston MW, Purkis SJ (2011). Spatial analysis of the invasion of lionfish in the western Atlantic and Caribbean. Marine Pollution Bulletin.

[ref-25] Kimball ME, Miller JM, Whitfield PE, Hare JA (2004). Thermal tolerance and potential distribution of invasive lionfish (*Pterois volitans/miles* complex) on the east coast of the United States. Marine Ecology Progress Series.

[ref-26] Kindinger TL, Albins MA (2017). Consumptive and non-consumptive effects of an invasive marine predator on native coral-reef herbivores. Biological Invasions.

[ref-27] Kletou D, Hall-Spencer JM, Kleitou P (2016). A lionfish (*Pterois miles*) invasion has begun in the Mediterranean Sea. Marine Biodiversity Records.

[ref-28] Kulbicki M, Beets J, Chabanet P, Cure K, Darling E, Floeter SR, Galzin R, Green A, Harmelin-Vivien M, Hixon M, Letourneur Y, De Loma TL, McClanahan T, McIlwain J, MouTham G, Myers R, O’Leary JK, Planes S, Vigliola L, Wantiez L (2012). Distributions of Indo-Pacific lionfishes *Pterois* spp. in their native ranges: implications for the Atlantic invasion. Marine Ecology Progress Series.

[ref-29] Lesser MP, Slattery M (2011). Phase shift to algal dominated communities at mesophotic depths associated with lionfish (*Pterois volitans*) invasion on a Bahamian coral reef. Biological Invasions.

[ref-30] McGuire M, Hill J (2014). Invasive species of florida’s coastal waters: the Red Lionfish (*Pterois volitans*) and Devil Firefish (*P. miles*) (IFAS Publication SGEF 208).

[ref-31] Mehrtens CJ, Rosenheim B, Modley M, Young RS (2001). Reef morphology and sediment attributes, Roatan, Bay Islands, Honduras. Carbonates and Evaporites.

[ref-32] Meister HS, Wyanski DM, Loefer JK, Ross SW, Quattrini AM, Sulak KJ (2005). Further evidence for the invasion and establishment of *Pterois volitans* (Teleostei: Scorpaenidae) along the Atlantic Coast of the United States. Southeastern Naturalist.

[ref-33] Morris JA, Akins JL, Barse A, Cerino D, Freshwater DW, Green SJ, Muñoz RC, Paris C, Whitfield PE (2009). Biology and ecology of the invasive lionfishes, P*terois miles* and *Pterois volitans*. Proceedings of the Gulf and Caribbean Fisheries Institute.

[ref-34] Norse EA, Brooke S, Cheung W, Clark MR, Ekeland I, Froese R, Gjerde KM, Haedrich RL, Heppell SS, Morato T, Morgan L, Pauly D, Sumaila R, Watson R (2012). Sustainability of deep-sea fisheries. Marine Policy.

[ref-35] Nuttall MF, Johnston MA, Eckert RJ, Embesi JA, Hickerson EL, Schmahl GP (2014). Lionfish (*Pterois volitans* [Linnaeus, 1758] and *P. miles* [Bennett, 1828]) records within mesophotic depth ranges on natural banks in the Northwestern Gulf of Mexico. BioInvasions Records.

[ref-36] Pinheiro HT, Goodbody-Gringley G, Jessup ME, Shepherd B, Chequer AD, Rocha LA (2016). Upper and lower mesophotic coral reef fish communities evaluated by underwater visual censuses in two Caribbean locations. Coral Reefs.

[ref-37] Pitt JM, Trott TM (2015). Trapping lionfish in Bermuda, Part II: lessons learned to date.

[ref-38] Quattrini AM, Demopoulos AWJ, Singer R, Roa-Varon A, Chaytor JD (2017). Demersal fish assemblages on seamounts and other rugged features in the northeastern Caribbean. Deep-Sea Research Part I-Oceanographic Research Papers.

[ref-39] R Core Team R (2013). http://www.r-project.org.

[ref-40] Rocha LA, Rocha CR, Baldwin CC, Weigt LA, McField M (2015). Invasive lionfish preying on critically endangered reef fish. Coral Reefs.

[ref-41] Rogers AD (1994). The biology of seamounts. Advances in Marine Biology.

[ref-42] Rogers AD (2015). Environmental change in the deep ocean. Annual Review of Environment and Resources.

[ref-43] Schofield PJ (2009). Geographic extent and chronology of the invasion of non-native lionfish (*Pterois volitans* [Linnaeus 1758] and *P. miles* [Bennett 1828]) in the Western North Atlantic and Caribbean Sea. Aquatic Invasions.

[ref-44] Schofield PJ (2010). Update on geographic spread of invasive lionfishes (*Pterois volitans* [Linnaeus, 1758] and *P. miles* [Bennett, 1828]) in the Western North Atlantic Ocean, Caribbean Sea and Gulf of Mexico. Aquatic Invasions.

[ref-45] Slattery M, Lesser MP (2014). Allelopathy in the tropical alga *Lobophora variegata* (Phaeophyceae): mechanistic basis for a phase shift on mesophotic coral reefs?. Journal of Phycology.

[ref-46] Sutherland WJ, Clout M, Côté IM, Daszak P, Depledge MH, Fellman L, Fleishman E, Garthwaite R, Gibbons DW, De Lurio J, Impey AJ, Lickorish F, Lindenmayer D, Madgwick J, Margerison C, Maynard T, Peck LS, Pretty J, Prior S, Redford KH, Scharlemann JPW, Spalding M, Watkinson AR (2010). A horizon scan of global conservation issues for 2010. Trends in Ecology & Evolution.

[ref-47] Switzer TS, Tremain DM, Keenan SF, Stafford CJ, Parks SL, McMichael Jr RH (2015). Temporal and spatial dynamics of the lionfish invasion in the eastern Gulf of Mexico: perspectives from a broadscale trawl survey. Marine and Coastal Fisheries.

[ref-48] Tornabene L, Baldwin CC (2017). A new mesophotic goby, *Palatogobius incendius* (Teleostei: Gobiidae), and the first record of invasive lionfish preying on undescribed biodiversity. PLOS ONE.

[ref-49] USGS-NAS (2017). US Geological Survey Nonindigenous Aquatic Species Database. https://nas.er.usgs.gov.

[ref-50] Whitfield PE, Muñoz RC, Buckel CA, Degan BP, Freshwater DW, Hare JA (2014). Native fish community structure and Indo-Pacific lionfish *Pterois volitans* densities along a depth-temperature gradient in Onslow Bay, North Carolina, USA. Marine Ecology Progress Series.

[ref-51] Wright DJ (2005). Report of HURL Cruise KOK0510: submersible dives and multibeam mapping to investigate benthic habitats of Tutuila, American Samoa. Technical Report.

